# Hospitalisation from fractures in New Zealand octogenarians: LiLACS NZ

**DOI:** 10.1007/s11657-025-01528-1

**Published:** 2025-04-09

**Authors:** Catherine J. Bacon, Simon A. Moyes, Joanna Hikaka, Ruth Teh, Astrid E. A. Atlasz, Ngaire Kerse

**Affiliations:** 1 School of Nursing, Faculty of Medical and Health Sciences, University of Auckland, PO Box 92019, Auckland, 1142 New Zealand; 2 Orthosports North Harbour Ltd, Auckland, New Zealand; 3 Department of General Practice and Primary Health Care, Faculty of Medical and Health Sciences, University of Auckland, Auckland, New Zealand; 4 Faculty of Medical and Health Sciences, Centre for Co-Created Ageing Research, University of Auckland, Auckland, New Zealand; 5 Faculty of Medical and Health Sciences, Te Kupenga Hauora Māori, University of Auckland, Auckland, New Zealand

**Keywords:** Injury, Frailty, Bone, Osteoporosis, Elderly

## Abstract

***Summary*:**

Longitudinal data quantifying fracture rates beyond 80 years are lacking. Over 5 years, hospitalised fracture incidences increased by 85% in Māori and 73% in non-Māori octogenarians. However, while fracture-related hospital nights increased by 107% in non-Māori, they remained stable for Māori. Hospitalised fracture risk increases markedly with 5 years of advanced ageing.

**Purpose:**

Fractures become increasingly common in people beyond 80 years, the most serious resulting in hospitalisation. This study examines longitudinal changes in hospitalised fractures in octogenarians.

**Methods:**

Hospital discharge records from a cohort study of Māori aged 80–90 years and non-Māori turning 85 years (LiLACS NZ) were used to determine the incidence of hospitalised fractures for 5 years before and 5 years after enrolment.

**Results:**

Records were available for 378 Māori (aged 82.6 ± 2.8 years; mean ± SD) and 498 non-Māori (84.6 ± 0.5 years). In the 5 years prior to enrolment, 22 (5.8%) Māori and 43 (8.6%) non-Māori were hospitalised at least once for fracture, and 29 (7.7%) Māori and 62 (12.4%) non-Māori sustained hospitalised fractures in the 5 years following enrolment. Hospitalised fracture incidences were 1270/100,000 person-years in Māori and 2048/100,000 person-years in non-Māori before enrolment, increasing to 2345 (*P* = 0.02) and 3541 (P = 0.002) /100,000 person-years in the subsequent 5 years, respectively. Pelvis/femoral fractures accounted for almost half (47%) of fractures. Fracture-related hospital nights increased 107% in non-Māori (*P* < 0.0001), but remained stable for Māori, from before to after enrolment. Following enrolment, 21% of hospital nights spent by non-Māori women were fracture-related.

**Conclusion:**

In octogenarians, hospitalised fracture risk increased markedly with 5 years of ageing, almost doubling fracture-related hospitalisation time in non-Māori but having little effect on time spent in hospital for Māori. Projections of fracture burden in advanced age need to consider rapidly changing risk with small increases in age and differences between demographic groups.

**Supplementary Information:**

The online version contains supplementary material available at 10.1007/s11657-025-01528-1.

## Introduction

The risk of hip fracture increases approximately sixfold from 65 to 85 years of age [1], and the 85 + age group is rapidly expanding [2, 3]. For some ethnic groups, the rate of demographic ageing is much higher; for example, in New Zealand, the number of Māori aged over 85 years increased over threefold from 1996 to 2018 [4]. Major fractures in advanced age are often related to osteoporosis and usually result in hospitalisation causing significant global disability and cost [5]. Of these, hip fractures are the most costly; extending beyond the initial hospital visit, due to the heightened risk of a subsequent fragility fracture and fracture-related complications [6]. One international estimate of direct hospital costs exceeded US $10,000 per hip fracture, but other health and social care costs in the subsequent year were over four times this figure [7]. Non-hip osteoporotic fractures are also costly, estimated, in Portugal, at around €3,000 per person for the year after a wrist, vertebral or other non-hip fracture [8]. In addition to economic costs, major fractures adversely impact quality-of-life [9] and are associated with threefold increases in 1-year mortality [10, 11]. Compared to major fractures, those classed as minor may not require hospitalisation, have approximately half the subsequent mortality rate and are associated with a less negative impact on quality of life [12].

Understanding fracture epidemiology is important for projecting fracture rates to estimate likely future resourcing implications, particularly with the ageing demographic in the next two decades. Fracture rates are hard to predict as they depend on availability and access to community preventative interventions and treatment and lifestyle factors such as physical activity modes and levels, diet, smoking and alcohol [13]. Other difficulties in predicting fracture rates and costs are due to demographic differences in risk with ethnicity and increasing age. A recent New Zealand study of unintentional injury in people aged 50 years and above showed differing injury risk with advancing age in Māori compared to non-Māori, with Māori at higher risk below 70 years, and increasing in risk less sharply above 80 years [14]. Despite the growing importance in an ageing population, few longitudinal studies report the incidence and nature of hospital fractures in advanced age or how these change across the ninth decade of life. Furthermore, despite increasing longevity, there is a distinct lack of data on fracture rates in Indigenous populations. Relevant to many Indigenous peoples, the association between socioeconomic inequities and fragility fracture incidence has recently been highlighted [15].

The primary aim of this study was to examine the effect of ageing on the epidemiology of fracture-related hospitalisations in advanced age. Using hospital records from a cohort of indigenous (Māori) and non-indigenous (non-Māori) octogenarians in New Zealand, we compared fracture rates, injury sites, hospitalisation time and fracture determinants in the 5 years prior to an index enrolment date with those in the 5 years after enrolment.

## Methods

### Study population

This study reports a prospective analysis of data from ‘Te Puāwaitanga o Ngā Tapuwae Kia ora Tonu. Life and Living in Advanced Age: a Cohort Study in New Zealand (LiLACS NZ)’. A cohort of 421 Māori (58% women) aged 80–90 years and 516 non-Māori (54% women) aged 85 years were recruited after a systematic invitation of people eligible by age in the Bay of Plenty region in New Zealand in 2010. Ethnicity was self-identified; if participants identified as Māori or as Māori and another ethnicity, they were included in the Māori cohort. Almost all, 99%, non-Māori were of European heritage. Details of recruitment show a 56% engagement rate for Māori, with a good representation of the regional Māori population, and a 59% engagement rate for non-Māori with an overrepresentation of men [16]. A broad range of measures were gathered at baseline, with methods of assessment previously described [17, 18]. Here, we report data from the core questions only, completed at baseline by almost all participants [18] and fractures from routinely collected national data, from participants who gave permission for access. All participants provided written, informed consent to take part in the cohort study, which was approved by the New Zealand Health and Disability Ethics Committee (NTX/09/09/088 and NTX/10/12/127), and 93% additionally gave permission for their medical records to be accessed [18].

### Measures

Publicly funded hospitalisation discharge diagnoses, ascertained from the New Zealand Ministry of Health, were coded with the International Classification of Diseases, 10th revision (ICD-10) fracture diagnosis codes. We identified bone fracture diagnoses: ICD-10 codes S02, S12, S22, S32, S42, S52, S62, S72, S82, S92, T02 and T12, excluding fractures caused by malignancy during the period from 5 years before the first interview in the study to 5 years following this date. Hospitalised fracture incidence was calculated separately for Māori men, Māori women, non-Māori men and non-Māori women as the number of fractures divided by the cumulative follow-up time in person-years, accounting for death. For each group, the number of nights spent in the hospital was also adjusted for person-years of follow-up.

At baseline (Wave 1), a questionnaire that included sociodemographic, education, health status and health-related questions was administered by interview. New Zealand Deprivation Index [19] was determined based on the address at inception, to approximate area socioeconomic deprivation. Residential care was ascertained at baseline. Falls were ascertained by self-report as 12-month recall at baseline.

Self-rated health, a 5-point scale from ‘poor’ to ‘excellent’, was ascertained from a single item from the SF-12 [20]. Depressive symptoms were defined as a positive response to either or both of two items: ‘During the past month have you often been bothered by feeling down, depressed or hopeless?’ and ‘During the past month have you often been bothered by little interest or pleasure in doing things?’ [21]. Eyesight and hearing disability were assessed via a 5-point Likert response (‘not at all’ to ‘extremely’) to items asking how these interfered with day-to-day functioning. Functional status was assessed from activities able to be done using a subset of the Nottingham Extended Activities of Daily Living questionnaire [22]: walking indoors, feeding yourself, making self a hot drink, taking hot drinks from one room to another, own housework, own shopping, full clothes wash, and using telephone, supplemented by three additional activities [23]: personal care, toileting, getting in and out of bed. These items were selected based on the frequency of reported difficulty from a feasibility study [24]. The 11 items were each scored from 0 if not done, 1 if done with help or 2 if done on their own, giving a maximum score of 22.

### Analysis

Analyses were performed using SAS™ software, version 9.4 (TS1M5) for Windows [SAS Institute Inc. Cary, NC, USA]. Descriptive statistics summarised the sample characteristics, prevalence of associated factors and outcomes. Bivariate associations between health variables and the occurrence of any fracture hospitalisation during the study period were analysed using independent *t*-tests for continuous and chi-square analysis for categorical demographic and health-related variables. Logistic regression models predicting any fracture hospitalisation were applied separately for all Māori, all non-Māori, all men and all women, including all available demographic and health-related variables as predictors of future fracture hospitalisation for 5 years after enrolment.

Analyses of the time between entry to the study and first fracture hospitalisation during the study period (survival analyses) were undertaken using Cox’s proportional hazard models. This approach was used due to anticipation that a high proportion would not sustain a fracture during follow-up; their time to first fracture is assumed in the model to be greater than the follow-up period.

## Results

A total of 937 participants (421 Māori, 516 non-Māori; 523 women, 414 men) were enrolled in LiLACS NZ (Wave 1) in 2010 [18]. Characteristics of the 876 (93.4% of) participants (162 Māori men, 216 Māori women, 233 non-Māori men and 265 non-Māori women) who gave permission for access to administrative health records are shown in Table 1. Māori (82.6 ± 2.8 years) were younger than non-Māori (84.6 ± 0.5 years) by design of the study and fewer Māori (40% compared to 48% non-Māori) reported having had a fracture in their lifetime (*P* = 0.03). The only gender differences were that women were more likely than men to have never smoked (*P* < 0.001) and men were more likely than women to have been recently hospitalised (*P* = 0.02) and to report hearing problems (*P* = 0.0002).

**Table 1:** Characteristics at baseline (Wave 1) by gender/ethnicity

	Māori		Non-Māori		*P* (ethnicity)	*P* (gender)
	Men	Women	Men	Women		
Sample size	162	216	233	265		
Age (years)	82.5 ± 2.8	82.7 ± 2.7	84.6 ± 0.5	84.6 ± 0.5	< 0.0001	0.48
High deprivation (decile 8–10)	97 (60)	151 (70)	90 (39)	115 (43)	< 0.0001	0.03
Education					< 0.0001	0.20
Basic (none/primary)	51 (32)	56 (26)	44 (19)	38 (15)	< 0.0001^e^	0.07^e^
Secondary	92 (58)	119 (56)	123 (54)	163 (63)		
Post-secondary	15 (9)	39 (17)	64 (27)	64 (23)		
Residential care	8 (5)	19 (9)	15 (6)	27 (10)	0.47	0.04
Hospitalised (last 12 months)	77 (48)	72 (33)	84 (36)	89 (34)	0.13	0.02
Smoking status					0.07	< 0.0001
Never	53 (34)	109 (51)	76 (33)	176 (67)	0.01^f^	< 0.0001^f^
Current	13 (8)	25 (12)	14 (6)	10 (4)		
Past	92 (58)	78 (37)	141 (61)	78 (30)		
Self-rated health^a^					0.79	0.72
Excellent	11 (7)	21 (10)	16 (7)	18 (7)	0.56^ g^	0.55^ g^
Very good	51 (32)	63 (29)	75 (32)	90 (34)		
Good	51 (32)	84 (39)	92 (40)	87 (33)		
Fair	41 (26)	37 (17)	36 (16)	60 (23)		
Poor	5 (3)	10 (5)	12 (5)	8 (3)		
Depressive symptoms^b^					0.20	0.64
Both	25 (16)	36 (17)	29 (13)	35 (14)		
Either	30 (19)	46 (22)	39 (17)	45 (18)		
Neither	127 (81)	168 (79)	190 (83)	211 (82)		
Eyesight problems^c^	23 (15)	45 (21)	43 (19)	52 (20)	0.66	0.21
Hearing problems^c^	57 (37)	60 (28)	74 (32)	56 (22)	0.08	0.002
Functional status^d^	8.8 ± 2.7	9.1 ± 3.0	9.2 ± 2.2	9.1 ± 2.6	0.31	0.57
Previous fracture	48 (48)	52 (34)	84 (45)	108 (51)	0.03	0.75
Falls (last 12 months)					0.07	0.18
None	106 (67)	137 (65)	143 (62)	151 (57)	0.054^ h^	0.26^ h^
1	21 (13)	34 (16)	50 (22)	48 (18)		
2 or 3	23 (15)	28 (13)	19 (8)	42 (16)		
> 3	8 (5)	13 (6)	19 (8)	23 (9)		

Data are mean ± SD for continuous variables and frequency (percentage of ethnicity/gender group) for categorical variables

^a^Rated using 5-point scale from ‘poor’ to ‘excellent’ in response to a single ‘general-health’ item from the SF-12 [20]

^b^Positive response to either or both of two items: ‘during the past month have you often been bothered by feeling down depressed or hopeless?’ and ‘during the past month have you often been bothered by little interest or pleasure in doing things?’ [21]

^c^Rated via 5-point Likert responses (‘not at all’ to ‘extremely’) to items asking how these interfered with day-to-day functioning [17]

^d^Assessed from activities able to be done using a subset of the Nottingham Extended Activities of Daily Living questionnaire [22]: walking indoors, feeding yourself, making self a hot drink, taking hot drinks from one room to another, own housework, own shopping, full clothes wash, and using telephone, supplemented by three additional activities [23]: personal care, toileting, getting in and out of bed. These items were selected based on frequency of reported difficulty from a feasibility study [24]. The 11 items were each scored from 0 if not done, 1 if done with help, or 2 if done on own, giving a maximum score of 22

^e^Primary or none versus higher education level

^f^Never versus ever smoked

^g^Fair or poor health rating versus higher

^h^None versus any falls

Average follow-up time after enrolment was less than 5 years, 4.7 ± 2.1 years, varying between gender-ethnicity groups from 4.1 ± 2.1 and 4.6 ± 2.1 years in Māori and non-Māori men to 4.8 ± 2.0 and 5.1 ± 1.9 years in Māori and non-Māori women. In the period following enrolment, 383 participants (41%) died. Mortality rates were higher in men than women (47% versus 36%, *P* < 0.001) and in Māori compared to non-Māori (45% versus 37%, *P* = 0.006), being much higher for Māori men than in other groups (55% versus 38%). Neither pre-enrolment fracture nor pre-enrolment hospitalisation rates predicted mortality.

Fracture-related hospitalisation incidence, overall and by body location, for each ethnicity-gender group is reported in Table 2. Hospitalised fracture rates were higher for non-Māori and increased significantly for both Māori (*P* = 0.02) and non-Māori (*P* = 0.002) in the 5-year follow-up period, compared to before enrolment, most markedly for non-Māori women (*P* = 0.01).

**Table 2:** Hospitalised-fracture incidence in the 5 years prior to and following study enrolment

		Māori men (*n* = 162)	Māori women (*n* = 216)	Non-Māori men (*n* = 233)	Non-Māori women (*n* = 265)			Total (*n* = 876)
5 years before enrolment		Age 75–90 y (mean 80.1 y)		Age 80–85 y (mean 82.1 y)				
Number of fractures								
None	151 (93.2)		205 (94.9)	217 (93.1)	238 (89.8)		811 (92.6)	
1	11 (6.8)		10 (4.6)	14 (6.0)	21 (7.9)		56 (6.4)	
2	0 (0)		0 (0)	2 (0.9)	6 (2.3)		8 (0.9)	
3	0 (0)		1 (0.5)	0 (0)	0 (0)		1 (0.1)	
Total fractures	11		13	18	33		75	
Incidence (/100,000 p-y)	1358		1204	1545	2491		1712	
Fracture locations								
Skull		0 (0)	1 (8)	0 (0)	1 (3)			2 (3)
Spine		2 (17)	0 (0)	0 (0)	3 (9)			5 (6)
Ribs/sternum		3 (25)	0 (0)	3 (15)	2 (6)			8 (10)
Clavicle/scapula/humerus		0 (0)	0 (0)	5 (25)	3 (9)			8 (10)
Lower arm/wrist		1 (8)	0 (0)	1 (5)	8 (24)			10 (13)
Hands/fingers		1 (8)	0 (0)	0 (0)	1 (3)			2 (3)
Pelvis/femur		3 (25)	9 (69)	10 (50)	14 (42)			36 (46)
Lower leg/ankle		2 (17)	2 (15)	0 (0)	1 (3)			5 (6)
Feet/toes		0 (0)	1 (8)	1 (5)	0 (0)			2 (3)
5 years after enrolment		Age 80–95 y (mean 85.1 y)		Age 85–90 y (mean 87.1 y)				
Number of fractures								
None	148 (91.4)		201 (93.1)	212 (91.0)	224 (84.5)		785 (89.6)	
1	14 (8.6)		11 (5.1)	19 (8.2)	35 (13.2)		79 (9.0)	
2	0 (0)		3 (1.4)	1 (0.4)	5 (1.9)		9 (1.0)	
3	0 (0)		1 (0.5)	1 (0.4)	1 (0.4)		3 (0.3)	
Total fractures		14	20	24		48	106	
Incidence (/100,000 p-y)		2419	2296	2624		4292	3043	
Skull		0 (0)	0 (0)	0 (0)		2 (4)	7 (2)	
Spine		3 (21)	0 (0)	2 (8)		3 (6)	8 (7)	
Ribs/sternum		1 (7)	4 (19)	5 (21)		6 (11)	16 (14)	
Clavicle/scapula/humerus		2 (14)	2 (10)	0 (0)		11 (21)	15 (13)	
Lower arm/wrist		0 (0)	2 (10)	2 (8)		9 (17)	13 (12)	
Hands/fingers		0 (0)	2 (10)	0 (0)		0 (0)	2 (2)	
Pelvis/femur		7 (50)	10 (48)	14 (58)		22 (42)	53 (47)	
Lower leg/ankle		1 (7)	1 (5)	0 (0)		0 (0)	2 (2)	
Feet/toes		0 (0)	0 (0)	1 (4)		0 (0)	1 (1)	

Data are frequency (percentage within each ethnicity-gender group) of people, except as indicated for Total fractures and Incidence. Fracture location numbers include multiple fracture locations sustained in the same incident. Therefore, the sum of fracture location incidents may be greater than the total number of incidents specified

*p-y* person-years

Pelvic or femoral fractures accounted for almost half the fractures before (46.2%) and after (47.3%) enrolment and increased proportionately more for men in the 5-year follow-up (Table 2). Upper body fractures, including the shoulder girdle, upper limb and chest but excluding head and spinal injuries, were also commonly reported before and after enrolment (35.9% and 41.1% of fractures, respectively) and increased markedly for women.

Prior to enrolment, non-Māori women spent more fracture-related nights in the hospital than other groups (428 nights/1000 person-years of follow-up, versus an average of 275 nights/1000 person-years in the other three groups; Table 3). Hospital nights per 1000 person-years doubled in non-Māori from 5 years before to 5 years after enrolment, increasing by 92% (*P* < 0.0001) in women and 111% (*P* < 0.0001) in men, but did not significantly increase in Māori men (3.7% higher) or Māori women (0.8% higher) In the follow-up period, fracture-related stays accounted for 11.3% and 21.0% of all hospital stays in non-Māori men and women, respectively, but only 6.4% of all hospital stays in Māori.

**Table 3:** Fracture-related nights spent in hospital in the 5 years prior to and following study enrolment

	Māori men (*n* = 162)	Māori women (*n* = 216)	Non-Māori men (*n* = 233)	Non-Māori women (*n* = 265)	Total (*n* = 876)
5 years before enrolment	Age 75–90 y (mean 80.1 y)		Age 80–85 y (mean 82.1 y)		
Fracture hospital nights	241	277	315	567	1400
Fracture nights/1000 p-y	298	256	270	428	320
All hospital nights	2475	2366	2917	3406	11,164
Percent of all hospital nights	9.7	11.7	10.8	16.6	12.5
5 years after enrolment	Age 80–95 y (mean 85.1 y)		Age 85–90 y (mean 87.1 y)		
Fracture hospital nights	179	225	476	1012	1892
Fracture nights/1000 p-y	309	258	520	905	543
All hospital nights	2760	3565	4218	4822	15,365
Percent of all hospital nights	6.5	6.3	11.3	21.0	12.3

*p-y* person-years

From the range of demographic and health-related independent variables assessed, only self-reported health at baseline (*P* = 0.04) and falling in the previous 12 months (*P* = 0.03 to 0.04) were significantly associated with any hospitalisation for fracture in the 5 years following recruitment (Table 4).

**Table 4:** Bivariate associations of baseline characteristics with hospitalised fracture: any versus none for all fractures over 5 years post-inception

	Māori men		Māori women		Non-Māori men		Non-Māori women		*P*
	Any (*n* = 14)	None (*n* = 148)	Any (*n* = 15)	None (*n* = 203)	Any (*n* = 21)	None (*n* = 212)	Any (*n* = 41)	None (*n* = 224)	Overall
Age	82.7 ± 2.8	82.5 ± 2.8	83.1 ± 2.8	82.7 ± 2.7	84.8 ± 0.4	84.6 ± 0.5	84.5 ± 0.5	84.6 ± 0.5	0.59
High deprivation (Dec 8–10)	7 (50)	90 (61)	10 (67)	143 (70)	4 (19)	86 (41)	17 (41)	98 (44)	0.11
Education									0.98
Basic (none/primary)	6 (50)	45 (31)	3 (20)	53 (27)	3 (14)	41 (20)	6 (15)	32 (15)	0.89^a^
Secondary	5 (42)	87(60)	8 (53)	111 (56)	11 (52)	112 (54)	28 (68)	135 (62)	
Post-secondary	1 (8)	14 (10)	4 (27)	33 (17)	7 (33)	55 (26)	7 (17)	52 (24)	
Residential care	2 (14)	6 (4)	2 (13)	17 (9)	0 (0)	15 (7)	3 (7)	24 (11)	0.81
Hospitalised (last 12 m)	4 (30)	73 (49)	7 (47)	65 (32)	10 (48)	74 (35)	13 (32)	76 (34)	0.72
Smoking status									0.16
Never	6 (46)	47 (32)	7 (50)	102 (52)	9 (43)	67 (32)	27 (66)	149 (67)	0.37^b^
Current	2 (15)	11 (8)	4 (29)	21 (11)	3 (14)	11 (5)	0 (0)	10 (4)	
Past	5 (38)	87 (60)	3 (21)	75 (38)	9 (43)	132 (63)	14 (34)	64 (29)	
Self-reported health									0.23
Excellent	1 (8)	10 (7)	0 (0)	21 (11)	0 (0)	16 (8)	1 (2)	17 (8)	0.04^c^
Very good	5 (38)	46 (32)	6 (40)	57 (29)	7 (33)	68 (32)	15 (37)	75 (34)	
Good	4 (31)	47 (32)	4 (27)	80 (40)	9 (43)	83 (40)	12 (29)	75 (34)	
Fair	3 (23)	38 (26)	1 (7)	36 (18)	4 (19)	32 (15)	11 (27)	49 (22)	
Poor	0 (0)	5 (3)	4 (27)	6 (3)	1 (5)	11 (5)	2 (5)	6 (3)	
Depressive symptoms									0.27^d^
Both	2 (15)	23 (16)	5 (33)	31 (16)	4 (19)	25 (12)	5 (13)	30 (14)	
Eyesight problems	3 (23)	20 (14)	2 (13)	43 (21)	6 (29)	37 (18)	10 (25)	42 (19)	0.27
Hearing problems	6 (46)	51 (36)	3 (20)	57 (29)	7 (33)	67 (32)	7 (18)	49 (22)	0.75
Functional status	8.5 ± 2.0	8.8 ± 2.7	8.5 ± 3.5	9.2 ± 3.0	9.3 ± 1.2	9.2 ± 2.3	9.3 ± 2.0	9.1 ± 2.7	0.79
Prior falls (last 12 m)									0.02
None	8 (62)	98 (68)	5 (36)	132 (67)	9 (43)	134 (64)	23 (56)	128 (57)	0.03^e^
1	3 (23)	18 (12)	3 (21)	31 (16)	6 (29)	44 (21)	7 (17)	41 (18)	
2 or 3	1 (8)	22 (15)	4 (29)	24 (12)	1 (4.8)	18 (9)	8 (20)	34 (15)	
> 3	1 (8)	7 (5)	2 (14)	11 (6)	5 (24)	14 (7)	3 (7)	20 (9)	

Data are mean ± SD or frequency (percent) for any fracture versus no fracture

^a^Primary/none versus other education

^b^Never versus ever smoking

^c^Excellent/very good/good versus fair/poor self-reported health

^d^Both or Geriatric Depression Scale (from full questionnaire) > 5 versus none

^e^None versus any falls

Few of the available variables were independently significant predictors of fracture hospitalisation in logistic regression models for all Māori, all non-Māori, all men and all women (shown in Table 5; full model in Online Resource 1). In Māori, current smokers had over six times greater odds of being hospitalised for fracture than past smokers (*P* = 0.02). For men, high deprivation was related to lower fracture odds (*P* = 0.03), and prior falls to higher odds (*P* = 0.03). For women, only ethnicity predicted fracture hospitalisation (*P* = 0.04).

**Table 5:** Odds ratios from logistic regression analyses of the occurrence of any fracture hospitalisation in the 5 years following recruitment

	Odds ratio [95% confidence interval]	*P*
All Māori (*n* = 355)		
Smoking status		0.02
Never	1.98 [0.71 to 5.54]	
Current	6.09 [1.68 to 22.10]	
Past	Reference	
All non-Māori (*n* = 474)	No independent determinants	
All men (*n* = 377)		
High deprivation (decile 8–10)	0.38 [0.16 to 0.91]	0.03
Smoking status		0.054
Never	1.97 [0.87 to 4.48]	
Current	4.03 [1.17 to 13.89]	
Past	Reference	
Prior falls (past 12 months)		0.03
None	0.16 [0.04 to 0.63]	
1	0.38 [0.09 to 1.52]	
2 or 3	0.12 [0.02 to 0.83]	
> 3	Reference	
All women (*n* = 452)		
Ethnicity (Māori versus non-Māori)	0.43 [0.20 to 0.94]	0.04

The following independent variables were included in models for each group: gender (for ethnicity models), ethnicity (for gender models), age, high deprivation (decile 8–10 versus other), education category, residential care, hospitalised in last 12 months, smoking status, self-reported health, depressive symptoms, eyesight problems, hearing problems, functional status, prior falls (none reference). Only those that attained statistical significance (including one borderline result) are shown in the table. Online Resource 1 shows parameters for all variables in the model

Like the logistic regression analysis, there were few important predictors in the analysis of time to first fracture (shown in Table 6; full model in Online Resource 2). Only better self-reported health for Māori (*P* = 0.0017) and fewer than three falls for men (*P* = 0.0057) were significantly related to reduced fracture hazard.

**Table 6:** Cox’s proportional hazard ratio survival analyses of time to first fracture after inception

	Hazard ratio (95% confidence interval)	*P*
All Māori (*n* = 316*)*		
Smoking status		0.07
Current	2.67 (0.89 to 7.35)	
Past	0.62 (0.25 to 1.48)	
Never	Reference	
Self-reported health		0.0017
Excellent	0.05 (0.002 to 0.53)	
Very good	0.29 (0.05 to 1.62)	
Good	0.13 (0.03 to 0.76)	
Fair	0.12 (0.03 to 0.62)	
Poor	Reference	
All non-Māori (*n* = 422)		
Gender (men versus women)	0.62 (0.37 to 1.04)	0.08
All men (*n* = 343)		
High deprivation (deciles 8–10)	0.51 (0.24 to 1.03)	0.08
Falls (last 12 m)		0.0057
None	0.17 (0.06 to 0.56)	
1	0.44 (0.15 to 1.51)	
2 or 3	0.18 (0.04 to 0.77)	
> 3	Reference	
All women (*n* = 395)		
Ethnicity (Māori versus non-Māori)	2.15 (1.09 to 4.62)	0.06
Self-reported health		0.08
Poor	7.56 (1.45 to 57.94)	
Fair	4.07 (1.10 to 26.46)	
Good	1.86 (0.51 to 11.93)	
Very good	2.72 (0.79 to 17.14)	
Excellent	Reference	

The following independent variables were included in models for each group: gender (for ethnicity models), ethnicity (for gender models), age, high deprivation (decile 8–10 versus other), education category, residential care, hospitalised in last 12 months, smoking status, self-reported health, depressive symptoms, eyesight problems, hearing problems, functional status, prior falls (none reference). Only those that attained statistical significance (including five borderline results) are shown in the table. Online Resource 2 shows parameters for all variables in the model

## Discussion

These results highlight the significant burden of fractures in octogenarians, accounting for over 300 hospital nights per thousand octogenarians annually, and an eighth of all hospital stays. Approximately one in 10 non-Māori aged 85–90 years (99% of European heritage) and one in 15 Māori aged 80–95 years could expect to be hospitalised for fracture over a 5-year period. Moreover, by comparing rates in the 5 years before study enrolment with those in the 5 years following, our findings show a 73% increase in person-years of hospitalised fracture incident rate from age 85 to age 90 years in non-Māori and a 78% increase in Māori, even though Māori were on average 2 years younger. In non-Māori, fracture-related hospital nights (per person-year) approximately doubled over the same period, though there was a negligible increase in hospital nights for Māori. From 85–90 years, fractures accounted for over a fifth of total hospital nights in non-Māori women and over a tenth in non-Māori men. A strength of this study is the completeness of follow-up and ascertainment of all hospitalisations using unique identifier matching.

Fracture rates in our cohort are comparable to previous studies, particularly when taking into account the declining rates over this century reported in some studies [25–27]. Most studies that have reported fracture rates by age and gender focus only on hip fractures. For men aged 80–90 years, these range from 689 to 1730: 1730 in Canada (from 1985 to 2005) [28], 1327 in Brazil (in 2011) [29], 1077 in Italy (2002 to 2008) [30], 1500 in the USA (1985 to 2005), [25], 850 in France (femoral neck only in 2016) [31], 775 in the Netherlands (1972 to 1987) [32] and 689 in Japan (in 2004) [33], all per 100,000 person-years. For 80- to 90-year-old women, hip fracture rates in the same studies range from 1450 to 3200: 3200 in Canada [28], 2328 in Brazil [29], 2006 in Italy [30], 2500 in the US [25], 1450 in France [31], 1550 in the Netherlands [32] and 1791 in Japan [33]. We report rates of pelvic and femoral fractures in non-Māori of 1147/100,000 person-years for men and 1424/100,000 person-years for women (2005 to 2015), similar to European comparators and consistent with a recent report from the Australia & New Zealand Hip Fracture Registry (ANZHFR), showing femoral neck fracture rates from 2018 to 2020 of around 700/100,000 for those aged 80–85 years increasing to 1600/100,000 for those 85–90 years [34]. The slightly younger (average 2 years) Māori men and women had hip fracture rates approximately a third lower than non-Māori men and women (775/100,000 person-years and 966/100,000 person-years, respectively). In contrast, ANZHFR data showed no difference in age-corrected fracture rates between Māori and non-Māori for all 9432 fractures sustained in those over 50 years [34].

Fewer studies have reported rates of all hospitalised fractures. Rates in non-Māori men and women here were 2085 and 3392/100,000 person-years, respectively, and 1189 and 1750/100,000 person-years for Māori men and women, respectively. A UK study including only osteoporotic fractures sustained between 2002 and 2018 reported lower rates of 954 and 2255/100,000 person-years for men and women, respectively [35], and rates in Germany for all fractures (including those not hospitalised) in 2019 were higher at 2646 and 4927/100,000 person-years for men and women, respectively [36]. Whilst recent studies show large increases in osteoporotic fracture rates above 85 years of age, compared to the 5- to 10-year age bracket below this [35, 37], unlike the current study, these are based on cross-sectional data and may thus be affected by cohort differences.

Pelvic and femoral fractures (mainly hip) had the highest incidence for all ethnicity-gender groups in both time periods, making up almost half of the fracture admissions. The number of pelvic and femoral fracture incidents increased by 50% with 5 years of ageing in non-Māori (by 40% in men and 57% in women) but more than doubled for Māori men. Hip fractures are commonly associated with osteoporotic bone loss and tend to be the costliest fractures [27]. Upper trunk (rib/sternum) and upper limb fractures (including shoulder girdle) were also common hospitalised fractures in all four demographic categories, varying in the 10 years studied from 23.8% of all fractures in Māori women to 45.7% in non-Māori women. Although 44% of all fractures in adults ≥ 20 years in France were upper trunk and limb fractures, the pattern of fractures changed over the life course; the proportion of upper body fractures declined through the sixth to eighth decades of life, and femoral neck fractures were the most prominent at 82 years [31]. Here, we show that upper body fractures remained common in the 80 s, with incidence increasing > 2.5-fold over 5 years in women (Māori and non-Māori combined).

Because of the economic and social burden of fractures and ageing populations, predicting and planning for future fracture rates is important. Projections are complicated by changes in fracture rates over time that result from changing age demographics, time-period and cohort effects [28, 38]. Absolute (non-age-adjusted) hip and fragility fracture incidence is increasing in many countries with ageing populations, due to larger numbers of people reaching advanced age [30, 36]. In contrast, several large North American studies and a Finnish study have reported declining rates of age-specific hip fractures for periods between the 1970s and 2019 [13, 25, 26, 38], though the opposite trend has been noted in the Netherlands from 1972 to 1987 [32] and Japan from 1987/1988 to 2004 [33]. Another US study reported a declining age-adjusted hip fracture rate that bottomed out during the early to mid-2010s [27]. Projections of future rates could be affected by small demographic differences between cohorts. Our data show that in octogenarians, 5 years of ageing can greatly increase fracture rates, though this rate of increase may not be stable across a broader age range.

In our prospective regression analyses (follow-up for the 5 years after enrolment), few independent variables were identified as significant predictors of hospitalised fracture occurrence. Māori women had less than half the odds of a hospitalised fracture compared to non-Māori. For men, living in the least deprived area reduced the odds of fracture by a similar margin, in accordance with evidence from the UK where fracture risk increased more across the deprivation spectrum for men than women [35]. Prior falls were a significant risk factor only for men, perhaps suggesting that into their 80 s, neuromuscular factors become a more important determinant of hospitalised fracture in men than women. In support of this possibility, two cohort studies of older men and women show longer chair-stand time [39] and inability to perform a standing balance test [40] were associated with falls in men but not women. In Māori, smoking increased fracture risk by sixfold, and poor self-reported health increased fracture hazard. Smoking is a known risk factor for osteoporotic fractures [41], and reduced smoking rates have been implicated in falling age-adjusted hip fracture incidence between 1970 and 2010 [38].

Our study has reported characteristics of fracture-related hospitalisations between Māori and non-Māori octogenarians. Fractures cause a moderate number of hospitalisations for Māori and culturally safe preventative measures are warranted [42]. Of note, despite a 78% and 91% increase in hospitalised fracture incident rates in Māori men and women from the 5 years before to 5 years after enrolment, fracture-related hospital nights per person-year increased in the same period by only 3.7% and 0.8%, respectively. The reasons for this are unclear. Because the increases were adjusted for follow-up in each group, this result is not due to differences in mortality rate; a recent NZ study confirms no difference in injury-related mortality between older Māori and non-Māori [14]. Māori may be experiencing fractures in the community and either not attending hospital or staying there for shorter durations than non-Māori. Furthermore, the proportion of all-cause hospital nights attributable to fracture reduced over time for Māori. This is likely to be due to the increasing prevalence with ageing of other conditions, such as cardiovascular disease, diabetes complications and cancer for Māori, relative to fractures. Our study highlights the importance of disaggregating data by ethnicity in epidemiological studies to better understand where appropriate interventions should be targeted to achieve equitable health outcomes [14].

A limitation of this study is that the duration of hospital stay for a fracture may have been influenced by frailty and other comorbidities, thus not reflecting the true hospital burden of fractures alone. Because this is a relatively small cohort, we were unable to determine predictors of hospitalised fractures in each of the four gender/ethnicity groups separately. Recent investment in national registries, both the ANZHFR, established in 2013 with patient-level data added from 2015, and the Australia & New Zealand Fragility Fracture Registry, established in 2022 may allow nuanced age and ethnicity analyses in the future [43]. This study may not have included all fractures. Community fractures, likely of less severity, are not included. Inequities in access to primary care for Māori may influence hospitalised fracture rates in this group [44]. In addition, we did not assess patient-reported outcomes resulting from these fractures to understand the holistic impact of fractures.

## Conclusion

In this longitudinal study of octogenarians, hospitalised fractures increased markedly with 5 years of ageing, particularly in non-Māori women. Approximately 1 in 10 non-Māori participants were hospitalised for fracture in the latter half of their 80 s, thus fractures are a major cause of hospitalisation in this group and represent a significant burden in terms of nights spent in hospital. Projections of fracture burden in advanced age need to consider rapidly changing risk with small increases in age.

## Supplementary Information

Below is the link to the electronic supplementary material.

Supplementary file1 (PDF 212 KB)

Supplementary file2 (PDF 232 KB)

## Data Availability

Please direct requests for data to the corresponding author.
