# Supplementary material for: Hospitalisation from fractures in New Zealand octogenarians: LiLACS NZ
Source: Arch Osteoporos. 2025 Apr 9;20(1):48. doi: 10.1007/s11657-025-01528-1 (PMC11982168; doi:10.1007/s11657-025-01528-1)
Supplement: Supplementary file 1 — Supplementary file1 (PDF 212 KB) [file 11657_2025_1528_MOESM1_ESM.pdf]

Hospitalisation from fractures in New Zealand octogenarians: LiLACS NZ

Catherine J Bacon, Simon A Moyes, Joanna Hikaka, Ruth Teh, Astrid EA Atlasz, Ngaire Kerse

Correspondence to: Dr Catherine J Bacon, School of Nursing, University of Auckland, PO Box 92019, Auckland 1142, New Zealand. Ph +649-923-1060; Email: c.bacon@auckland.ac.nz

Online Resource 1

Full Models for Logistic Regression Analyses

Supplementary Table 1 Full Models for Logistic Regression Analyses

	Odds ratio (95% confidence interval)	p
All Māori	N = 355	
Sex (M v F)	1.65 (0.66, 4.13)	0.29
Age	1.00 (0.84, 1.19)	0.97
High deprivation (deciles 8 – 10)	0.52 (0.21, 1.32)	0.17
Education		0.61
post-secondary	0.89 (0.24, 3.24)	
secondary	0.61 (0.22, 1.70)	
basic (none/primary)	Ref	
Residential care (Y v N)	3.69 (0.74, 18.28)	0.11
Hospitalised in the last 12 months	0.92 (0.35, 2.43)	0.87
Smoking status		0.023
current	3.07 (0.93, 10.14)	
past	0.51 (0.18, 1.41)	
never	Ref	
Self-reported health		0.16
poor	10.50 (0.67, 163.77)	
fair	1.28 (0.11, 14.89)	
good	2.69 (0.28, 25.98)	
very good	3.97 (0.43, 36.66)	
excellent	Ref	
Depressive symptoms mild to severe versus none	1.66 (0.54, 5.13)	0.38
Eyesight problems	0.78 (0.22, 2.75)	0.69
Hearing problems	0.79 (0.29, 2.13)	0.64
Functional status	1.06 (0.87, 1.29)	0.58
Falls (last 12m)		0.10
>3	4.71 (0.83, 26.87)	
2 or 3	2.34 (0.69, 7.89)	
1	3.62 (1.14, 11.49)	
None	Ref	

	Odds ratio (95% confidence interval)	p
All Non-Māori	N = 474	
Sex (M v F)	0.62 (0.33, 1.17)	0.14
Age	1.09 (0.63, 1.88)	0.75
High deprivation (deciles 8 – 10)	0.74 (0.41, 1.36)	0.34
Education		0.58
post-secondary	0.81 (0.31, 2.11)	
secondary	1.19 (0.53, 2.64)	
basic (none/primary)	Ref	
Residential care (Y v N)	0.30 (0.05, 1.95)	0.21
Hospitalised in the last 12 months	1.15 (0.62, 2.14)	0.65
Smoking status		0.92
current	0.89 (0.47, 1.68)	
past	1.08 (0.28, 4.18)	
never	Ref	
Self-reported health		0.53
poor	3.50 (0.27, 45.72)	

fair	5.82 (0.71, 47.63)	
good	4.04 (0.51, 31.84)	
very good	4.39 (0.56, 34.47)	
excellent	Ref	
Depressive symptoms mild to severe versus none	0.92 (0.38, 2.22)	0.84
Eyesight problems	1.29 (0.65, 2.57)	0.47
Hearing problems	0.95 (0.48, 1.85)	0.87
Functional status	0.99 (0.82, 1.19)	0.91
Falls (last 12m)		0.34
>3	2.32 (0.89, 6.04)	
2 or 3	1.11 (0.47, 2.65)	
1	1.46 (0.70, 3.06)	
None	Ref	

	Odds ratio (95% confidence interval)	p
All Men	N = 377	
Ethnicity (Māori vs non-Māori)	1.34 (0.54, 3.38)	0.53
Age	1.06 (0.85, 1.32)	0.63
High deprivation (deciles 8 – 10)	0.38 (0.16, 0.91)	0.029
Education		0.56
post-secondary	1.05 (0.35, 3.16)	
secondary	0.67 (0.27, 1.69)	
basic (none/primary)	Ref	
Residential care (Y v N)	1.10 (0.16, 7.47)	0.93
Hospitalised in the last 12 months	0.94 (0.41, 2.13)	0.88
Smoking status		0.054
current	0.51 (0.22, 1.15)	
past	2.04 (0.59, 7.10)	
never	Ref	
Self-reported health		0.78
poor	1.02 (0.04, 23.40)	
fair	2.28 (0.23, 22.09)	
good	2.84 (0.33, 24.21)	
very good	2.87 (0.34, 24.04)	
excellent	Ref	
Depressive symptoms mild to severe versus none	1.39 (0.44, 4.38)	0.58
Eyesight problems	1.37 (0.53, 3.57)	0.51
Hearing problems	0.90 (0.40, 2.04)	0.81
Functional status	1.04 (0.84, 1.29)	0.71
Falls (last 12m)		0.033
>3	6.39 (1.58, 25.83)	
2 or 3	0.79 (0.16, 3.81)	
1	2.40 (0.94, 6.13)	
None	Ref	

	Odds ratio (95% confidence interval)	p
All Women	N = 452	
Ethnicity (Māori vs non-Māori)	0.43 (0.20, 0.94)	0.036
Age	1.03 (0.84, 1.27)	0.75
High deprivation (deciles 8 – 10)	1.04 (0.55, 1.96)	0.91
Education		0.67

post-secondary	0.74 (0.25, 2.13)	
secondary	1.09 (0.47, 2.50)	
basic (none/primary)	Ref	
Residential care (Y v N)	0.81 (0.17, 3.79)	0.79
Hospitalised in the last 12 months	1.08 (0.56, 2.09)	0.82
Smoking status		0.74
current	0.97 (0.50, 1.90)	
past	1.57 (0.47, 5.18)	
never	Ref	
Self-reported health		0.11
poor	17.85 (1.63, 195.18)	
fair	4.51 (0.54, 37.94)	
good	3.52 (0.44, 28.28)	
very good	4.42 (0.55, 35.36)	
excellent	Ref	
Depressive symptoms mild to severe versus none	1.08 (0.45, 2.58)	0.86
Eyesight problems	0.92 (0.42, 2.02)	0.84
Hearing problems	0.87 (0.40, 1.88)	0.72
Functional status	1.01 (0.85, 1.21)	0.89
Falls (last 12m)		0.57
>3	1.68 (0.56, 5.06)	
2 or 3	1.61 (0.72, 3.59)	
1	1.46 (0.64, 3.30)	
None	Ref	

The following independent variables were included in each model: gender (for ethnicity models) ethnicity (for gender models), age, high deprivation (decile 8-10 versus other), education category, residential care, hospitalised in last 12 months, smoking status, self-reported health, depressive symptoms, eyesight problems, hearing problems, functional status, prior falls (none reference).
