# Supplementary material for: Hospitalisation from fractures in New Zealand octogenarians: LiLACS NZ
Source: Arch Osteoporos. 2025 Apr 9;20(1):48. doi: 10.1007/s11657-025-01528-1 (PMC11982168; doi:10.1007/s11657-025-01528-1)
Supplement: Supplementary file 2 — Supplementary file2 (PDF 232 KB) [file 11657_2025_1528_MOESM2_ESM.pdf]

Hospitalisation from fractures in New Zealand octogenarians: LiLACS NZ

Catherine J Bacon, Simon A Moyes, Joanna Hikaka, Ruth Teh, Astrid EA Atlasz, Ngaire Kerse

Correspondence to: Dr Catherine J Bacon, School of Nursing, University of Auckland, PO Box 92019, Auckland 1142, New Zealand. Ph +649-923-1060; Email: c.bacon@auckland.ac.nz

Online Resource 2

Full Models for Cox's Proportional Hazard Ratio Survival Analyses

Supplementary Table 2 Full Models for Cox's Proportional Hazard Ratio Survival Analyses

	Hazard ratio (95% confidence interval)	p
All Māori	N = 316	
Sex (M v F)	1.44 (0.62, 3.32)	0.39
Age	1.04 (0.89, 1.20)	0.61
High deprivation (deciles 8 – 10)	0.52 (0.23, 1.14)	0.10
Education		0.36
post-secondary	0.81 (0.24, 2.38)	
secondary	0.51 (0.21, 1.24)	
basic (none/primary)	Ref	
Residential care (Y v N)	3.30 (0.73, 12.44)	0.12
Hospitalised in the last 12 months	1.15 (0.49, 2.58)	0.75
Smoking status		0.07
current	2.67 (0.89, 7.35)	
past	0.62 (0.25, 1.48)	
never	Ref	
Self-reported health		0.0017
poor	19.92 (1.87, 495.65)	
fair	2.43 (0.36, 48.43)	
good	2.67 (0.45, 51.38)	
very good	5.73 (1.07, 107.06)	
excellent	Ref	
Depressive symptoms mild to severe versus none	1.15 (0.39, 2.96)	0.82
Eyesight problems	0.59 (0.16, 1.72)	0.38
Hearing problems	0.78 (0.31, 1.79)	0.60
Functional status	0.90 (0.79, 1.05)	0.14
Falls (last 12m)		0.18
>3	2.88 (0.52, 12.33)	
2 or 3	1.64 (0.49, 4.60)	
1	3.03 (1.06, 7.97)	
None	Ref	

	Hazard ratio (95% confidence interval)	p
All Non-Māori	N = 422	
Sex (M v F)	0.62 (0.37, 1.04)	0.08
Age	0.93 (0.60, 1.46)	0.77
High deprivation (deciles 8 – 10)	1.07 (0.65, 1.76)	0.78
Education		0.32
post-secondary	0.85 (0.37, 1.96)	
secondary	1.34 (0.72, 2.76)	
basic (none/primary)		
Residential care (Y v N)	1.23 (0.26, 4.98)	0.78
Hospitalised in the last 12 months	1.14 (0.69, 1.87)	0.63
Smoking status		0.94

current	1.26 (0.29, 3.76)	
past	1.04 (0.61, 1.77)	
never	Ref	
Self-reported health		0.12
poor	3.07 (0.47, 25.03)	
fair	4.58 (1.27, 29.37)	
good	2.16 (0.62, 13.68)	
very good	2.95 (0.86, 18.51)	
excellent	Ref	
Depressive symptoms mild to severe versus none	0.93 (0.43, 1.80)	0.83
Eyesight problems	1.30 (0.72, 2.25)	0.39
Hearing problems	1.25 (0.72, 2.11)	0.42
Functional status	0.93 (0.78, 1.12)	0.45
Falls (last 12m)		0.24
>3	1.97 (0.83, 4.26)	
2 or 3	1.44 (0.69, 2.80)	
1	1.68 (0.89, 3.05)	
None	Ref	

	Hazard ratio (95% confidence interval)	p
All Men	N = 343	
Ethnicity (Māori vs non-Māori)	0.86 (0.41, 1.96)	0.71
Age	1.06 (0.87, 1.28)	0.58
High deprivation (deciles 8 – 10)	0.51 (0.24, 1.03)	0.08
Education		0.96
post-secondary	1.03 (0.38, 2.70)	
secondary	0.91 (0.43, 2.04)	
basic (none/primary)		
Residential care (Y v N)	1.23 (0.17, 5.78)	0.84
Hospitalised in the last 12 months	1.08 (0.53, 2.13)	0.83
Smoking status		0.16
current	2.94 (0.90, 8.26)	
past	0.93 (0.47, 1.86)	
never	Ref	
Self-reported health		0.51
poor	1.15 (0.04, 35.39)	
fair	3.31 (0.55, 63.58)	
good	3.26 (0.63, 59.91)	
very good	4.56 (0.91, 83.08)	
excellent	Ref	
Depressive symptoms mild to severe versus none	1.52 (0.57, 3.53)	0.37
Eyesight problems	1.23 (0.54, 2.57)	0.61
Hearing problems	1.10 (0.56, 2.11)	0.76
Functional status	0.91 (0.78, 1.09)	0.28
Falls (last 12m)		0.0057
>3	6.08 (1.79, 17.90)	

2 or 3	1.10 (0.25, 3.36)	
1	2.70 (1.22, 5.79)	
None	Ref	

	Hazard ratio (95% confidence interval)	p
All Women	N = 395	
Ethnicity (Māori vs non-Māori)	2.15 (1.09, 4.62)	0.06
Age	1.09 (0.90, 1.30)	0.35
High deprivation (deciles 8 – 10)	1.11 (0.64, 1.92)	0.73
Education		0.52
post-secondary	0.76 (0.31, 1.86)	
secondary	1.15 (0.59, 2.43)	
basic (none/primary)	Ref	
Residential care (Y v N)	2.34 (0.61, 8.42)	0.28
Hospitalised in the last 12 months	1.12 (0.64, 1.93)	0.70
Smoking status		0.60
current	1.75 (0.51, 4.65)	
past	0.97 (0.53, 1.70)	
never	Ref	
Self-reported health		0.08
poor	7.56 (1.45, 57.94)	
fair	4.07 (1.10, 26.46)	
good	1.86 (0.51, 11.93)	
very good	2.72 (0.79, 17.14)	
excellent	Ref	
Depressive symptoms mild to severe versus none	0.86 (0.39, 1.75)	0.73
Eyesight problems	1.00 (0.50, 1.84)	0.99
Hearing problems	1.15 (0.59, 2.11)	0.69
Functional status	0.96 (0.82, 1.15)	0.73
Falls (last 12m)		0.49
>3	1.31 (0.47, 3.12)	
2 or 3	1.65 (0.81, 3.17)	
1	1.33 (0.63, 2.65)	
None	Ref	

The following independent variables were included in each model: gender (for ethnicity models) ethnicity (for gender models), age, high deprivation (decile 8-10 versus other), education category, residential care, hospitalised in last 12 months, smoking status, self-reported health, depressive symptoms, eyesight problems, hearing problems, functional status, prior falls (none reference).
